# On the Lithontriptic Properties of Soda

**Published:** 1820-01

**Authors:** Ed. Sutliffe

**Affiliations:** Queen-street


FOR THE LONDON MEDICAL AND PHYSICAL JOURNAL.

A CASE exhibiting the solvent properties of the sal sodæ, recently occurred in the following calculous complaint: Mr. F. W. a near relative of mine, at the west-end of the town, had for a considerable time past laboured under renal and cystic complaints, who resorted to a vast variety of means, without deriving any important relief. During the few last months he took large quantities of sal sodæ; and, although much stony matter was evacuated, he became so impatient as to entreat Mr. Astley Cooper to remove it by lithotomy, which was in a few days afterwards performed, with his accustomed skill. I received the different portions of the calculi; and, such was the character of the offending matter, that a considerable part appeared to have been corroded by a solvent, so as to be broken down with great facility: other portions of its calculous basis, lodged in cysts, were hard, and could not be tangible by the solvent; but, had an equal exposure to its influence been attainable, I feel persuaded that the necessity for the operation would have been superseded.

ED. SUTLIFFE.

*Queen-street; November 9th,* 1819.

